# Index to the Transactions of the Epidemiological Society of London

**Published:** 1855-12

**Authors:** 


### INDEX.

Bowel diseases in the Crimean expedition, 55

Cholera in Tynemouth, 25; in Frankfort, 66; premonitory diarrhœa of, 72

Crimean expedition, bowel diseases in, 55

Diarrhœa premonitory of cholera, 72

Epidemics and contagion, 96

Epidemiological Society, origin and objects of, 1; report of council, 4; quarterly reports of, 51, 82, 97

Frankfort-on-the-Maine, cholera in, 66

Grainger, Mr. R. D., animal effluvia and their effects on health, 83

Greenhow, Dr. E. H., cholera in Tynemouth, 25

Mortality, comparative, of towns and rural districts, 16

Nurses, plan of providing, for the labouring classes, 10

Riecke, Dr., views on epidemics and contagion, 96

Smart, Dr. W., bowel diseases in the Crimean expedition, 55

Snow, Dr., comparative mortality of large towns and rural districts, 16

Todd, Mr. G., premonitory diarrhœa of cholera, 72

Towns and rural districts, comparative mortality of, 16

Tynemouth, cholera in, 25

Varrentrapp, Dr. G., cholera in Frankfort, 66

